# Association of DNA damage response gene polymorphisms with treatment response and prognosis in acute myeloid leukemia

**DOI:** 10.7150/jca.134391

**Published:** 2026-07-01

**Authors:** Amin Zhang, Hanyang Wu, Wancheng Liu, Daoxin Ma

**Affiliations:** 1 Department of Hematology, Qilu Hospital of Shandong University, No.107, West of Wenhua Road, Jinan, 250012, Shandong, People's Republic of China.; 2 Department of Pediatrics, Qilu Hospital of Shandong University, No.107, West of Wenhua Road, Jinan, 250012, Shandong, People's Republic of China.; 3 Department of Clinical Laboratory, Qilu Hospital of Shandong University, No.107, West of Wenhua Road, Jinan, 250012, Shandong, People's Republic of China.; 4 Shandong Key Laboratory of Hematological Diseases and Immune Microenvironment, Qilu Hospital of Shandong University, No.107, West of Wenhua Road, Jinan, 250012, Shandong, People's Republic of China.; 5 Shandong Provincial Clinical Research Center for Hematological Diseases, Qilu Hospital of Shandong University, No.107, West of Wenhua Road, Jinan, 250012, Shandong, People's Republic of China.

**Keywords:** AML, SNPs, DDR pathway, treatment response

## Abstract

The potential functional Single Nucleotide Polymorphism (SNPs) of DNA Damage Response (DDR)-related genes may influence AML aggressiveness and clinical outcomes through regulating genomic stability. However, systematic evaluation of functionally relevant SNPs across the entire DDR cascade in AML remains limited. In our study, significant associations were identified across multiple DDR modules. In the damage sensing module, *MRE11* rs2155209 TC/CC genotypes were protective against hyperleukocytosis and associated with longer OS, while *NBS1* rs1805794 CG/GG genotypes increased hyperleukocytosis risk. In the signal transduction module, *CHEK1* rs521102 AA genotype was a risk factor for thrombocytopenia but unexpectedly associated with prolonged OS; *CHEK2* rs738722 TT genotype increased hyperleukocytosis risk; *RAD17* rs1045051 GT/GG genotype conferred a higher relapse risk; and *BRCA1* rs1799949 GA/AA genotype as a protective factor for high BM blast percentage. In the DNA repair module, *LIG3* rs3744356 TT genotypes predicted higher WBC counts and lower complete remission rate. Both *LIG3* rs3744356 TT genotype and *MGMT* rs2296675 GA genotype were associated with shorter OS (HR = 1.719, 95% CI = 1.082 -2.741, *p* = 0.022; HR = 1.511, 95% CI = 1.046 - 2.182, *p* = 0.028). SNPs in effector genes *TP53* and *CDKN1A* showed no significant associations. In summary, SNPs across the DDR pathway—particularly in damage sensing, signal transduction, and DNA repair modules—are significantly associated with AML clinical features, treatment response, and survival. Our findings provide a foundation for developing DDR-based polygenic risk models and support the potential integration of germline SNP profiling into precision medicine strategies for AML.

## Introduction

Acute myeloid leukemia (AML) is a common hematological malignancy driven by the abnormal accumulation of genomic mutations in hematopoietic stem/progenitor cells 1, 2. Although the combination of cytarabine and daunorubicin has been established as the conventional standard-intensity chemotherapy regimen for AML, approximately 20 - 40% of patients fail to achieve complete remission (CR) 3. The main mechanism of conventional chemotherapy is to induce DNA damage of AML cells, thereby triggering the cell apoptosis. The levels of DNA damage could influence the effects of chemotherapy 4, 5. Therefore, it is of importance to explore the intrinsic DNA damage of AML cells to evaluate the effects of chemotherapy.

Genetic variations have been reported to affect the chemosensitivity or DNA damage of AML cells at different aspects 6. For the somatic variations, acquired driver mutations directly lead to differentiation arrest and proliferative advantage acquisition in AML cells 7-9. Therefore, somatic mutations in *CEBPA*, *NPM1* and *FLT3* et al. have been incorporated into the risk stratification of AML by the European LeukemiaNet (ELN) guidelines, directly guiding prognostic assessment and treatment decisions 10. For the germline variations, single nucleotide polymorphisms (SNPs) constitute the genetic background underlying interindividual differences, subtly shaping physiological state by influencing mechanisms such as gene transcription, mRNA stability, or protein function. In particular, SNPs in key genes of the DNA damage response (DDR) pathway have been shown to play important roles in prostate cancer, breast cancer to process genotoxic drugs, thereby affecting the sensitivity to chemotherapy 11, 12. However, given that individual DDR SNPs have been sporadically studied in AML, a systematic, pathway-based evaluation covering the entire signaling cascade, from damage sensing to DNA repair execution, remains lacking.

In this study, we focus on a set of potentially functional SNPs within the DNA damage checkpoint pathway. These loci comprehensively cover the entire signaling cascade, ranging from damage sensing (e.g., sites related to MRE11, RAD50, and NBS1, components of the MRN complex), through signal transduction and amplification (e.g., CHEK2, CHEK1, RAD17), to the central decision point (the TP53 codon 72 polymorphism). Additionally, we include relevant loci for LIG3, which affects end-joining repair, and for the growth factor IGF1, which may interfere with the p53 pathway. This investigation aims to elucidate how this multi-gene SNP profile collectively shapes the clinical presentation of AML and modulates patient response and prognosis following DNA damage-based chemotherapy.

## Materials and Methods

### Study Population

A total of 245 patients with newly diagnosed AML were enrolled at Qilu Hospital of Shandong University between January 2010 and January 2023. Patients were excluded if they were: 1) under 14 years of age; 2) had received prior anti-leukemic therapy; 3) presented with other primary malignancies; 4) lacked available biological samples. All diagnoses were confirmed according to the National Comprehensive Cancer Network (NCCN) guidelines and the World Health Organization (WHO) classification. The cohort had a median age of 52 years (range: 15 - 79). All patients received induction chemotherapy consisting of an anthracycline combined with cytarabine. Written informed consent was obtained from each participant prior to inclusion in accordance with the Declaration of Helsinki, and the study was approved by the Institutional Ethics Committee of Qilu Hospital (Approval No. KYLL-202204-059). Basic information and clinical characteristics of the patients are summarized in **Table 1**.

### DNA extraction and SNP genotyping

Peripheral blood samples collected at the time of diagnosis were used for genomic DNA extraction. Mononuclear cells were first isolated by Ficoll-Hypaque density gradient centrifugation, and genomic DNA was subsequently extracted using a commercial kit (TianGen, Beijing, China) according to the manufacturer's protocol. DNA concentration and purity were assessed by spectrophotometry, after which aliquots were stored at -80°C until further processing. Candidate SNPs involved in DDR were curated for this study (**Table 2**). Selection and functional annotation of these SNPs were based on the dbSNP and UCSC databases. For each SNP, corresponding primers were developed using Agena Assay Designer 4.0. Three primers, one universal extension primer and two amplification primers, were synthesized per locus and purified via polyacrylamide gel electrophoresis (PAGE). Genotyping assays were conducted with approximately 20 ng of input DNA per sample. Detection was performed on a MassARRAY platform, followed by sample analysis using MALDI-TOF/TOF mass spectrometry. To ensure assay accuracy, 6 negative controls (no DNA template) and 6 positive controls (samples with known genotypes) were inter-spiked among the study samples. Additionally, 16 randomly selected samples were genotyped in duplicate across two independent assay panels, yielding a reproducibility rate of 99%.

### Statistical analysis

Hardy-Weinberg equilibrium (HWE) was assessed using the chi-square test. SNPs conforming to HWE (*p* > 0.05) and exhibiting a minor allele frequency (MAF) greater than 5% in the general population were retained for subsequent analysis. Genotyping data were evaluated under four genetic models: dominant, recessive, codominant, and allelic. To assess associations between SNP genotypes/alleles and clinical outcomes, we performed statistical analyses in two stages. First, categorical variables were screened using the chi-square test or Fisher's exact test, as appropriate. Second, univariate binary logistic regression models—adjusted for sex and age—were applied to calculate odds ratios (ORs) with corresponding 95% confidence intervals (95% CIs) and *p*-values. Given the exploratory nature of multiple SNP association testing across several genetic models and clinical endpoints, we applied the Benjamini-Hochberg procedure to control the false discovery rate (FDR). Adjusted *p*-values (q-values) were calculated for primary SNP-outcome associations. Findings with nominal *p* < 0.05 were reported, but emphasis was placed on associations that remained significant after FDR correction.

Survival analysis was performed using Kaplan-Meier curves with comparisons based on the log-rank test. Associations between SNPs and overall survival (OS) in AML patients were evaluated with the Cox proportional hazards model, yielding hazard ratios (HRs) and corresponding 95% CIs. Prognostic variables were initially screened via univariate Cox analysis, and significant factors were subsequently incorporated into a multivariate Cox model for further assessment.

A multivariate Cox regression model with backward selection was applied to assess the independent prognostic significance of the following covariates: age (< 60 vs. ≥ 60 years), ELN risk category (favorable, intermediate, adverse), white blood cell count at diagnosis (< 100 vs. ≥ 100 × 10⁹/L) and PLT count (< 50 vs. ≥ 50 × 10⁹/L) All statistical analyses were conducted using SPSS version 26.0 (IBM Corp., Chicago, IL, USA). A two-sided *p*-value < 0.05 was considered statistically significant.

## Results

### SNPs selection and study populations

The selected SNPs of genes associated with DR checkpoint are listed in **Table 1**. HWE and MAF were used for the initial screening of candidate SNPs. SNPs with *p* ≥ 0.05 in the HWE test and MAF ≥ 0.05 in the general population were selected for further analysis. Accordingly, two loci with a minor allele frequency (MAF) < 0.05 (*ATM* rs1801516 and *ATR* rs1802904) and two loci deviating from the Hardy-Weinberg equilibrium (HWE) (*HUS1* rs1056663 and *P21* rs1801270) were excluded from further analysis. A total of 245 patients were recruited in this study. The basic information, clinical characteristics and initial treatment response using cytarabine and doxorubicin of these patients were shown in **Table 2**.

### Association between bone marrow (BM) blasts at initial diagnosis

To further explore the value of these SNPs in AML, we first analyzed the relationship between SNPs and the percentage of BM blasts at initial diagnosis (**Table 3**). The proportion of BM blasts at initial diagnosis served as a surrogate for disease burden. A BM blast percentage of 70% or greater was defined as high, and below 70% as low. The* LIG3* rs3744356 and *BRCA1* rs1799949 under dominant models, were significantly associated with the percentage of BM blast at initial diagnosis (*p* < 0.05). After the adjustment of age and sex, the CT genotype under co-dominant model (OR = 2.114, 95% CI = 1.206-3.705, *p* = 0.009) and CT/TT genotype under dominant model (OR = 1.831, 95% CI = 1.087 - 3.082, *p* = 0.023) of *LIG3* rs3744356 tended to be a risk factor. In contrast, for *BRCA1* rs1799949, the GA genotype under the co-dominant model (OR = 0.556, 95% CI = 0.311 - 0.992, *p* = 0.048) and the GA/AA genotype under the dominant model (OR = 0.539, 95% CI = 0.312 - 0.933, *p* = 0.027) emerged as protective factors against a higher BM blast percentage at diagnosis.

### Association between peripheral blood characteristics and SNPs

Considering that peripheral blood characteristics might be related to SNPs, we analyzed the association of selected SNPs with WBC, HGB, and PLT in AML patients using the χ^2^ test or Fisher's exact test. We found the high WBC group consisted of patients with a WBC count equal to or greater than 100 × 10^9^/L, while the low WBC group included patients with a WBC count less than 100 × 10^9^/L. The high PLT group was defined as patients with PLT levels greater or equal to than 50 × 10^9^/L, while the low PLT group included patients with PLT levels less than 50 × 10^9^/L. The high HGB group included patients with an HGB level equal to or greater than 60 g/L, while the low HGB group included patients with an HGB level less than 60 g/L. As shown in **Table 3**,* MRE11* rs2155209 under the co-dominant and dominant models, *CHEK2* rs738722 under the co-dominant and recessive models, *LIG3* rs3744356 under the recessive model, and *NBS1* rs1805794 under the dominant model were significantly associated with AML patients with a high WBC count (*p* < 0.05). After adjusting for age and sex, the TC genotype of *MRE11* rs2155209 under the co-dominant model (OR = 0.29, 95% CI = 0.094 - 0.895, *p* = 0.031) and the TC/CC genotype under the dominant model (OR = 0.321, 95% CI = 0.114 - 0.899, *p* = 0.031) were identified as protective factors against high WBC count in AML patients. In contrast, the TT genotype under the codominant model and recessive model of *CHEK2* rs738722 are associated with an increased risk of high WBC count in AML. TT genotype in *LIG3* rs3744356 recessive model and CG/GG genotype in *NBS1* rs1805794 dominant model tended to be a risk factor for AML patients with a high WBC blasts at diagnosis (OR > 1, *p* < 0.05). However, no SNPs were found to be associated with HGB levels (*p* > 0.05). After adjustment of sex and age, the AA genotype of *CHEK1* in rs521102 is a risk of low PLT under recessive model (OR = 2.188, 95% CI = 1.03 - 4.648, *p* = 0.042) (**Table 4**).

### Polymorphisms are related to sensitivity to cytarabine/anthracycline-induced therapy in AML

We analyzed the associations between SNPs and sensitivity to cytarabine- and anthracycline-containing agents. Among the 245 non-M3 AML patients analyzed and underwent bone marrow cytomorphological assessment after the first course of induction with cytarabine- and anthracycline- containing regimens. Preliminary screening with the χ^2^ test or Fisher's exact test showed that rs3744356 in* LIG3* under the co-dominant and recessive model were significantly correlated with response to chemotherapy (*p* < 0.05). After adjustment of sex and age, and applying FDR correction, the TT genotype of rs3744356 were found to be significantly related to response to chemotherapy under the co-dominant (OR = 1.81, 95% CI = 1.16 - 2.28, *p* = 0.009) and recessive models (OR = 3.174, 95% CI = 1.376 - 7.323, *p* = 0.007), with a decrease in the CR group (**Table 5**).

### DDR-related polymorphisms are related to AML relapse

Preliminary screening using the χ^2^ or Fisher's exact test was conducted under three genetic models. As shown in **Table 6**, *RAD17* rs1045051 under co-dominant and dominant models were significantly associated with the AML relapse (*p* < 0.05). After adjustment of sex and age and applying FDR correction, the GT genotype of *RAD17* rs1045051 under co-dominant model (OR = 2.92, 95% CI = 1.347 - 6.327 *p* = 0.007) and GT/GG genotype under dominant model (OR = 2.433 95% CI = 1.216 - 4.869, *p* = 0.012) tended to be a risk factor for AML relapse.

### DDR-related polymorphisms are associated with AML overall survival

**Figure 1:**
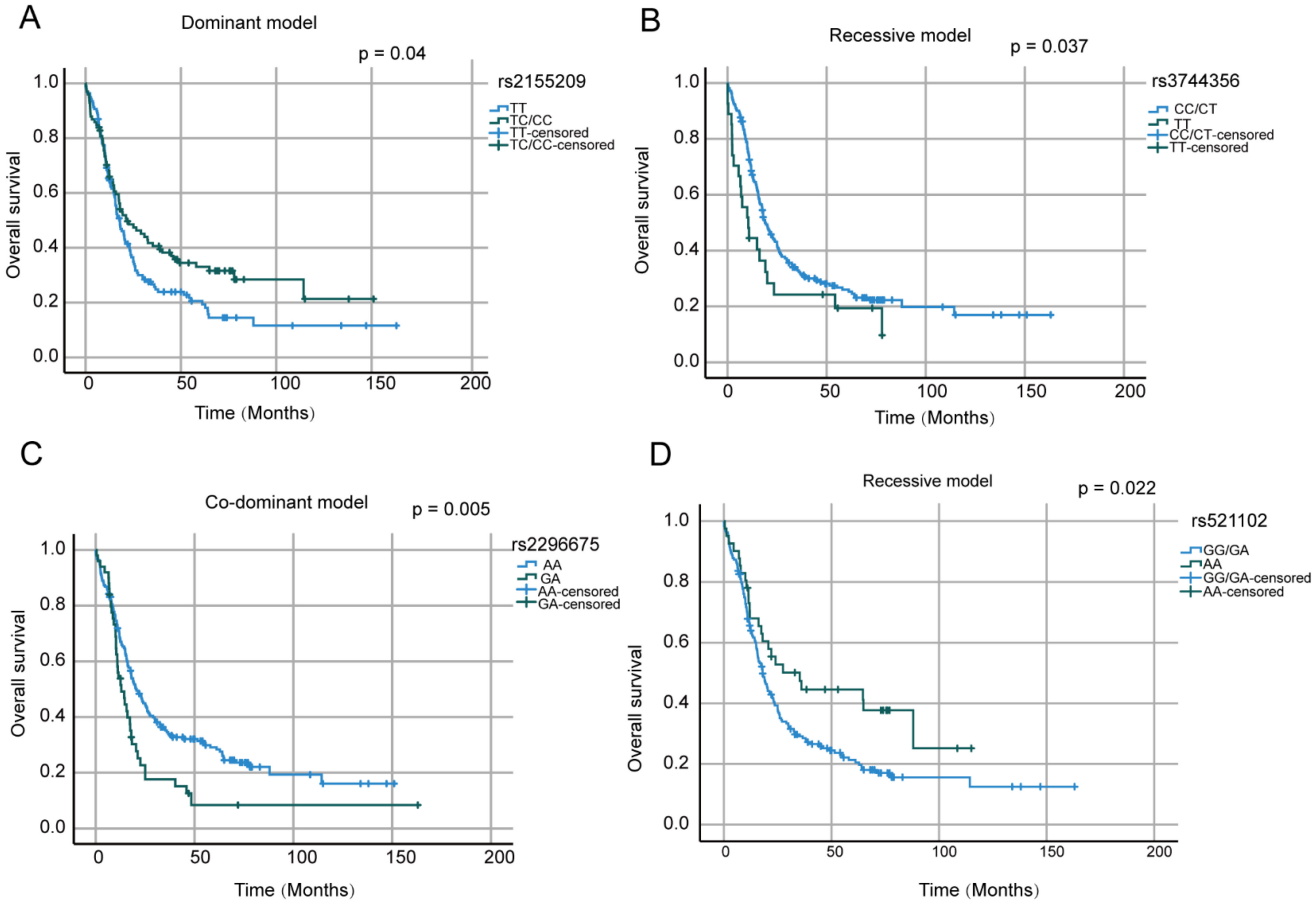
Overall survival of AML patient with SNPs of 4 DDR related genes. (A) Rs2155209 SNP and OS in dominant model; (B) Rs3744356 and OS in recessive model; (C) Rs2296675 and OS in co-dominant model; (D) Rs521102 and OS in recessive model.

We then analyzed the relationships between various SNPs and overall survival (OS) in AML patients. Kaplan-Meier analysis revealed an association between the genotype frequency of rs2155209 in *MRE11* and prognosis under a dominant model (*p* < 0.05). Specifically, AML patients carrying the TC/CC genotype exhibited significantly longer overall survival (OS) compared to those with the TT genotype. Kaplan-Meier screening revealed that rs3744356 in *LIG3* and rs521102 in *CHEK1* under recessive model and rs2296675 in *MGMT* under Co-dominant model were associated with prognosis (*p <* 0.05, **Figure 1**).

**Figure 2:**
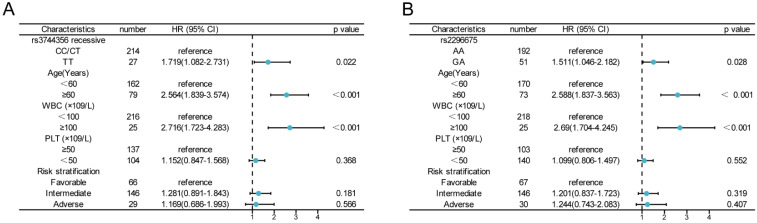
The impact of* LIG3* rs3744356 and *MGMT* rs2296675 on the outcomes of AML patients. (A) Multivariate cox regression analysis of rs3744356 recessive model and clinical characteristics associated with OS; (B) Multivariate cox regression analysis of *MGMT* rs2296675 co-dominant model and clinical characteristics associated with OS.

A Cox proportional hazards model with multivariate analysis for OS was used to analyze the above four SNPs, age, risk stratification, WBC and PLT count at diagnosis (**Figure 2**). In our study, age ≥ 60 years, WBC ≥100×10^9^/L had an independent negative impact on OS. Patients with the TT genotype of rs3744356 in *LIG3*, remained significantly associated with worse OS (HR = 1.719, 95% CI = 1.082 -2.741, *p* = 0.022). Similarly, GA genotype of rs2296675 in *MGMT* remained significantly associated with worse OS (HR = 1.511, 95% CI = 1.046 - 2.182, *p* = 0.028). These results demonstrated that the TT genotype of rs3744356 in *LIG3* and GA genotype of rs2296675 in *MGMT* is an independent risk prognostic factor for AML patients.

## Discussion

The development and progression of AML rely on acquisition of specific somatic mutations 13. SNPs in genes involved in the DDR pathway modulate genomic stability, thereby creating a favorable genetic background for the emergence of driver mutations and subsequent clonal evolution 13-15. In this study, we systematically evaluated potentially functional SNPs across 4 key modules of the DDR pathway—damage sensing, signal transduction, effector activation, and DNA repair—in patients with AML. Significant associations were identified between several SNPs and AML aggressiveness or clinical outcome, including variants in DNA damage sensing genes (*MRE11* rs2155209, *NBS1* rs1805794), signal transduction genes (*CHEK1* rs521102, *CHEK2* rs738722, *RAD17* rs1045051 and *BRCA1* rs1799949), and DNA repair genes (*LIG3* rs3744356 and *MGMT* rs2296675). In contrast, SNPs of DDR related genes in effector activation, such as *TP53* and *CDKN1A*, showed no significant association with AML. These findings suggest that the impact of germline genetic variation within the DDR pathway on AML may predominantly exert its influence through the integrated processes of damage recognition, signal amplification, and repair execution.

Why do gene SNPs within different modules of the DDR cascade exert distinct functional and clinical effects? The differential impact of SNPs across DDR modules likely reflects the hierarchical and context-dependent architecture of the DDR cascade. Variants in damage sensing genes may modulate the activation threshold of genomic surveillance, whereas SNPs in signal transduction genes influence checkpoint intensity and cell fate decisions. In contrast, polymorphisms in DNA repair genes directly determine repair capacity and thus chemotherapy responsiveness. Furthermore, the effects of germline SNPs may be masked in core effector genes such as TP53 by dominant somatic mutations. Given the high replication stress and genotoxic therapy exposure in AML, module-specific dependencies within the DDR network may account for the heterogeneous clinical associations observed.

The MRN ternary complex, composed of MRE11, RAD50 and NBS1, is one of the most critical damage sensors in the DDR pathway, playing a central role in recognizing DNA double-strand breaks, initiating signal transduction, and maintaining genomic stability 16, 17. Our study found that the TC/CC genotypes of the *MRE11* rs2155209 polymorphism served as protective factors against hyperleukocytosis in AML patients. Furthermore, survival analysis revealed that carriers of the *MRE11* rs2155209 variant alleles had a longer OS. Currently, research on *MRE11* rs2155209 in hematologic malignancies is limited, and studies in other cancers, such as bladder or breast cancer, have not identified this SNP as a protective factor against malignancy 18, 19. Functionally, rs2155209, located in the non-coding region of the *MRE11* gene, may upregulate *MRE11* expression by influencing mRNA splicing or translational efficiency, thereby promoting apoptosis of AML cells in response to chemotherapeutic agents. Conversely, the CG/GG genotypes of the *NBS1* rs1805794 polymorphism were identified as risk factors for hyperleukocytosis. Previous studies have reported inconsistent associations between *NBS1* rs1805794 and susceptibility to hematologic malignancies, potentially illustrating the functional heterogeneity of this locus across different tumor types and genetic backgrounds 20, 21. Rs1805794 is located in the BRCT domain of *NBS1*; the substitution of Gln for Glu may weaken the binding affinity between NBS1 and MRE11, thereby reducing the efficiency of DNA damage signal transduction.

The *CHEK1*, *CHEK2*, *RAD17* and *BRCA1* genes play key roles in the signal transduction and amplification module of the DDR pathway. Our study identified multiple significant associations within this module. For *CHEK1* rs521102, the AA genotype served as a risk factor for thrombocytopenia but was associated with prolonged OS, suggesting a complex role where this variant may influence platelet production while simultaneously enhancing chemotherapy sensitivity 22, 23. The CHEK2 rs738722 TT genotype has been identified as a risk factor for hyperleukocytosis, consistent with findings from multiple studies suggesting its potential association with poor cancer prognosis 24. This effect may occur through modulation of CHEK2 activation efficiency, thereby impacting downstream p53-dependent apoptotic pathways. For RAD17 rs1045051, the GT/GG genotype emerged as a risk factor for AML relapse, which might be explained by impaired checkpoint function that reduces the effective elimination of residual leukemic clones following chemotherapy. We also identified the *BRCA1* rs1799949 GA/AA genotype as a protective factor for high BM blast percentage. BRCA1 is a multifaceted tumor suppressor involved in both DNA damage signaling and homologous recombination repair 25, 26. Although rs1799949 is a synonymous variant located in exon 11, it may up-regulate BRCA1 expression through effects on mRNA stability or splicing efficiency, thereby modulating the efficiency of both checkpoint activation and downstream repair processes 27. Functionally, this variant could enhance the DNA damage response capacity of leukemic cells, preventing against more aggressive disease features.

In contrast, SNPs in the core effector genes *TP53* and *CDKN1A* showed no significant associations in our study, which may be caused by several factors. Somatic *TP53* mutations occur at high frequency in AML, particularly in cases with complex karyotypes 28, 29. The dominant-negative or loss-of-function effects of *TP53* mutations could easily overshadow any subtle influence of germline variants. Besides, the DDR signaling cascade contains considerable functional redundancy 30. The ATM/CHK2/p53 axis operates in parallel with other DNA damage checkpoints, and p21, encoded by *CDKN1A*, can be regulated through p53-independent and post-transcriptional or post-translational mechanisms, providing compensatory routes that buffer the impact of germline SNPs in single effector genes 31, 32. Additionally, because these pivotal tumor-suppressor genes are under strong purifying selection, common germline variants are likely to be functionally mild, further reducing their detectability in association studies of the present sample size 33.

The DNA repair module serves as the final executor of the DDR pathway, directly determining whether damaged cells undergo apoptosis or survive with genomic alterations 34. Our study identified significant associations of AML course and 2 key repair genes, *LIG3* and *MGMT*. For *LIG3* rs3744356, the TT genotypes were associated with multiple adverse clinical features, including elevated WBC counts, lower CR rate following induction chemotherapy, and shorter OS. LIG3 plays a critical role in base excision repair and alternative non-homologous end joining; functionally, rs3744356 may upregulate LIG3 expression by influencing mRNA splicing or stability, thereby enhancing the repair capacity of leukemic cells against chemotherapy-induced DNA damage and promoting drug resistance 35. For *MGMT* rs2296675, the variant allele was identified as a risk factor for shorter OS. MGMT directly repairs alkylating agent-induced DNA damage, and rs2296675, though a synonymous variant, may influence MGMT expression through codon usage bias or mRNA stability, potentially allowing damaged cells to survive and proliferate 36, ultimately leading to a poorer prognosis in AML. Currently, studies on both *LIG3* rs3744356 and *MGMT* rs2296675 in AML are limited; our findings provide the evidence linking these variants to clinical outcomes in AML. Together with our observations in the damage sensing and signal transduction modules, these results support the concept that genetic variation across the entire DDR cascade—from damage recognition to final repair—collectively shapes AML aggressiveness, treatment response, and patient survival.

In summary, this study systematically evaluated the association between a multi-gene SNP panel spanning the DDR pathway and AML aggressiveness and prognosis. Our findings highlight that germline variants in damage sensing, signal transduction, and DNA repair modules play significant roles in AML development and chemosensitivity, while the contribution of SNPs in effector genes such as *TP53* and *CDKN1A* may be limited in this context. Given the widespread accessibility of SNP genotyping assays and the relatively high frequency of these variants in the Chinese population, prospective evaluation of these SNPs in clinical laboratories is entirely feasible. However, several limitations should be acknowledged. The modest sample size may limit the statistical power for multivariate analyses and subgroup assessments. The use of dbSNP database allele frequencies as controls, rather than a matched healthy cohort, may introduce population stratification bias. Additionally, the precise molecular mechanisms by which these SNPs influence DDR function remain to be elucidated, and somatic mutation data were not integrated into the current analysis. Future studies with larger, independent cohorts and well-matched controls are warranted to validate our findings. Functional experiments are also needed to clarify how these variants modulate DDR pathway activity and ultimately influence AML progression and treatment response. Despite these limitations, our results provide a foundation for developing a DDR-based polygenic risk score model and open new avenues for integrating germline genetic profiling into precision medicine strategies for AML.

## Figures and Tables

**Table 1:** The selected genes and SNPs.

	Genes	SNPs	MAF	HWE (*p*-value)
DNA damage sensing	*MRE11*	rs2155209	0.237	0.602
	*NBS1*	rs1805794	0.425	0.663
	*RAD50*	rs2706347	0.155	0.598
Signal transduction	*ATM*	rs1801516	0.039	0.523
	*ATR*	rs1802904	0.040	0.336
	*CHEK1*	rs521102	0.384	0.077
	*CHEK2*	rs738722	0.279	0.757
	*RAD17*	rs1045051	0.368	0.744
	*BRCA1*	rs1799949	0.40	0.749
	*BARD1*	rs6435862	0.102	0.754
	*Hus1*	rs1056663	0.443	0.003
Effector activation	*TP53*	rs1042522	0.435	0.795
	*P21*	rs1801270	0.447	0.0009
DNA repair / Damage resolution	*LIG4*	rs1805388	0.194	0.72
	*LIG3*	rs3744356	0.323	0.602
	*LIG1*	rs20579	0.115	0.887
	*MGMT*	rs2296675	0.105	0.067
	*BRCA2*	rs9567552	0.312	0.068

**Table 2:** Clinical features of patients with AML.

Variable	Case n (%)
Gender	
Male	127 (51.8)
Female	118 (48.2)
Age (years, median, range)	
Median	52 (15 - 79)
< 60	172 (70.2)
≥ 60	73 (29.8)
WBC (×10^9^/L)	
Median	14.74 (0.48 - 351.95)
< 100	220 (89.8)
≥ 100	25 (10.2)
PLT (×10^9^/L)	
Median	43 (5 - 861)
≥ 50	104 (42.5)
< 50	141 (57.5)
HGB (g/L)	
Median	75 (27 - 136)
≥ 60	202 (82.4)
< 60	43 (17.6)
Risk stratification	
Favorable	68 (27.8)
Intermediate	147 (60.0)
Adverse	30 (12.2)
Response after 1 cycle of cytarabine- and anthracycline- containing induction therapy	
CR	151 (61.6)
No CR	94 (38.4)

**Table 3:** Association between SNPs and the percentage of BM blast of AML patients.

Gene	SNP	Model	Genotype	BM blast < 70%	BM blast ≥ 70%	χ^2^ test *p-*value	OR (95% CI)	Adjusted *p-*value
*LIG3*	rs3744356	Co-dominant	CC	57	55	0.02		
			CT	33	69		2.114(1.206-3.705)	0.009
			TT	13	14		1.087(0.465-2.54)	0.847
		Dominant	CC	57	55	0.019		
			CT/TT	46	83		1.831(1.087-3.082)	0.023
*BRCA1*	rs1799949	Co-dominant	GG	29	58	0.065		
			GA	58	62		0.556(0.311-0.992)	0.048
			AA	19	19		0.723(0.486-1.076)	0.11
		Dominant	GG	29	58	0.02		
			GA/AA	77	81		0.539(0.312-0.933)	0.027

**Table 4:** The relationship between peripheral blood characteristics and SNPs in AML patients at the first diagnosis.

Gene	SNP	Model	Genotype	WBC <100×10^9^/L	WBC ≥100×10^9^/L	χ^2^ test*p-*value	OR (95% CI)	Adjusted *p-*value
*MRE11*	rs2155209	Co-dominant	TT	122	19			
			TC	80	4	0.098	0.29 (0.094-0.895)	0.031
			CC	14	1		0.659 (0.231-1.879)	0.436
		Dominant	TT	122	19			
			TC/CC	94	5	0.032	0.321 (0.114-0.899)	0.031
*LIG3*	rs3744356	Recessive	CC/CT	195	19			
			TT	21	6	0.032	3.019 (1.078-8.454)	0.035
*CHEK2*	rs738722	Co-dominant	CC	118	9			
			CT	85	11	0.044	1.766 (0.697-4.478)	0.231
			TT	15	5		2.049 (1.104-3.803)	0.023
		Recessive	CC/CT	203	20			
			TT	15	5	0.041	3.336 (1.086-10.25)	0.035
*NBS1*	rs1805794	Dominant	CC	77	4			
			CG/GG	138	21	0.047	3.099 (1.019-9.421)	0.046
Gene	SNP	Model	Genotype	PLT ≥ 50×10^9^/L	PLT < 50×10^9^/L	χ^2^ test *p-*value	OR (95% CI)	Adjusted *p-*value
*CHEK1*	rs521102	Recessive	GG/GA	88	105			
			AA	11	30	0.027	2.188 (1.03-4.648)	0.042

**Table 5:** Association between SNP of and AML induction therapy response.

Gene	SNP	Model	Genotype	CR	No CR	χ^2^ test *p-*value	OR (95% CI)	Adjusted *p-*value
*LIG3*	rs3744356	Co-dominant	CC	72	40			
			CT	67	35	0.019	0.937 (0.531-1.653)	0.822
			TT	10	17		1.81 (1.16-2.28)	0.009
		Recessive	CC/CT	139	75	0.005		
			TT	10	17		3.174 (1.376-7.323)	0.007

**Table 6:** Association between SNPs and AML relapse.

Gene	SNP	Model	Genotype	Relapse	No relapse	χ^2^ test *p-*value	OR (95% CI)	Adjusted *p-*value
*RAD17*	rs1045051	Co-dominant	TT	56	26			
			GT	74	13	0.035	2.92 (1.347-6.327)	0.007
			GG	20	7		1.177 (0.703-1.971)	0.535
		Dominant	TT	56	26			
			GT/GG	94	20	0.021	2.433 (1.216-4.869)	0.012

## Data Availability

The data that support the findings of this study are available from the corresponding author upon reasonable request.
